# The economic burden of musculoskeletal disease in Korea: A cross sectional study

**DOI:** 10.1186/1471-2474-12-157

**Published:** 2011-07-13

**Authors:** In-Hwan Oh, Seok-Jun Yoon, Hye-Young Seo, Eun-Jung Kim, Young Ae Kim

**Affiliations:** 1 Department of Preventive Medicine, College of Medicine, Korea University, Seoul, Korea; 2 Graduate School of Korea University, Department of Public Health, Seoul, Korea; 3 Department of Nursing, Cheju Halla College, Jeju, Korea

## Abstract

**Background:**

Musculoskeletal diseases are becoming increasingly important due to population aging. However, studies on the economic burden of musculoskeletal disease in Korea are scarce. Therefore, we conducted a population-based study to measure the economic burden of musculoskeletal disease in Korea using nationally representative data.

**Methods:**

This study used a variety of data sources such as national health insurance statistics, the Korea Health Panel study and cause of death reports generated by the Korea National Statistical Office to estimate the economic burden of musculoskeletal disease. The total cost of musculoskeletal disease was estimated as the sum of direct medical care costs, direct non-medical care costs, and indirect costs. Direct medical care costs are composed of the costs paid by the insurer and patients, over the counter drugs costs, and other costs such as medical equipment costs. Direct non-medical costs are composed of transportation and caregiver costs. Indirect costs are the sum of the costs associated with premature death and the costs due to productivity loss. Age, sex, and disease specific costs were estimated.

**Results:**

Among the musculoskeletal diseases, the highest costs are associated with other dorsopathies, followed by disc disorder and arthrosis. The direct medical and direct non-medical costs of all musculoskeletal diseases were $4.18 billion and $338 million in 2008, respectively. Among the indirect costs, those due to productivity loss were $2.28 billion and costs due to premature death were $79 million. The proportions of the total costs incurred by male and female patients were 33.8% and 66.2%, respectively, and the cost due to the female adult aged 20-64 years old was highest. The total economic cost of musculoskeletal disease was $6.89 billion, which represents 0.7% of the Korean gross domestic product.

**Conclusions:**

The economic burden of musculoskeletal disease in Korea is substantial. As the Korean population continues to age, the economic burden of musculoskeletal disease will continue to increase. Policy measures aimed at controlling the cost of musculoskeletal disease are therefore required.

## Background

Musculoskeletal disease includes osteoarthritis, rheumatoid arthritis, osteoporosis, and low back pain. These diseases are public health concerns and have a heavy burden on society owing to their chronic characteristics and the associated severe pain. In addition, they also affect the psychological health of patient families and caregivers [1]. The burden of osteoarthritis was 2.5% of the total disability adjusted life years (DALYs) in high-income countries in 2001, where osteoarthritis was ranked as the tenth leading cause of DALYs [2]. A study conducted in 2004 determined that osteoarthritis accounts for 4.1% of the total years lost to disability (YLD), which ranks fifth among all diseases [3]. The importance of musculoskeletal disease is also evident from the results of studies conducted in individual nations. For example, in the USA, the self-recognized prevalence of arthritis was 30% among patients 18 years or older. In those 65 years or older group, the prevalence rate was even 55% [4]. Additionally, a burden of disease study in Singapore revealed that musculoskeletal disease accounted for 4.8% of total DALYs in 2004 and was the sixth leading cause of disease and injury behind neurological and sense disorders [5].

Korea is not an exception to this trend. In Korea, musculoskeletal disease follows cancer, cardiovascular disease, digestive disease, respiratory disease, and neuropsychiatric disease to be ranked seventh in total DALYs for major disease groups in 2002. Additionally, among musculoskeletal diseases, rheumatoid arthritis ranks 14^th ^in DALYs for males, while rheumatoid arthritis ranks fifth and osteoarthritis ranks eighth for females [6].

Musculoskeletal disease is also a significant economic burden. The economic cost of musculoskeletal disease in Canada accounted for 3.4% of the GDP in 1994, and it is estimated that the cost of musculoskeletal disease was 7.7% of the GDP in the USA, which reached $849 billion dollars in 2004 [7,8]. In Korea, musculoskeletal disease accounted for 28.2% of National Health Insurance Corporation (NHIC) inpatient and outpatient claims, 10.9% of NHIC benefits, and 0.27% of the GDP in 2008 [9,10]. The self-recognized prevalence of osteoarthritis, disc disorders, osteoporosis, and rheumatoid arthritis were reported to be 13.0%, 6.2%, 4.1%, and 2.2%, respectively, and the self-recognized population prevalence of musculoskeletal disease was 19,720 per 100,000 persons in adults in 2005. Additionally, 73.1% of women 65 years and older will have one or more types of musculoskeletal disease such as osteoarthritis, disc disorders, osteoporosis, or rheumatoid arthritis in their lifetime [11]. Another study estimated musculoskeletal disease prevalence using a questionnaire survey and reported the prevalence of osteoarthritis, rheumatoid arthritis, herniated discs, osteoporosis, spondyloarthropathy, and gouty arthritis to be 3.2%, 1.6%, 1.1%, 0.8%, 0.3%, and 0.1%, respectively. However, these results differ from those of previous studies [12,13].

The prevalence of most musculoskeletal diseases increase with age and are affected by lifestyle and habits such as physical activity and obesity [1]. Significant increases in the incidence and prevalence of musculoskeletal disease are expected as a result of global population aging and changing lifestyles. As a result, the United Nations and the World Health Organization endorsed 2000-2010 as the "Bone and Joint Decade" [1].

Social changes in Korea such as population aging, increasing rates of obesity will soon make musculoskeletal disease a central public concern [14,15]. Adequate policies will be required to facilitate the prevention and management of musculoskeletal disease. Studies of the pathophysiology, epidemiology, and burden of musculoskeletal disease will be needed to inform policy makers. Measuring the economic costs associated with musculoskeletal disease facilitates an understanding of where the greatest economic costs occur during treatment and care, and this knowledge will in turn guide the creation of public health policy [16]. However, the use of data from other countries is limited in that the prevalence of musculoskeletal disease varies by country and race. Therefore, data from Korea-specific studies would be the most helpful for application to the Korean population; however, such studies are limited [13], especially studies which focus on the economic burden of musculoskeletal disease.

In this study, the importance of musculoskeletal disease in terms of economic burden was determined using a number of data sources such as NHIC statistics, the Korea Health Panel study, and cause of death reports generated by the Korea National Statistical Office (NSO).

## Methods

This study measures the economic burden of musculoskeletal disease using the prevalence approach, which estimates the economic cost of all cases in a given period [17]. The costs of disease are classified as direct, indirect, and intangible [18]. Direct costs are those associated with direct medical care and direct non-medical care. Direct medical costs are defined as the costs due to diagnosis, treatment, continuing care, rehabilitation and terminal care. Direct non-medical costs are costs incurred through the use of non-health care resources such as transportation, legal and court costs, cost of relocating, and informal care. In contrast, indirect costs include the opportunity costs associated with hospitalization and outpatient visits, as well as those due to premature death. Intangible costs include the psychological suffering resulting from the disease.

In this study, economic burden was measured using the methodology outlined in previous studies [19,20]. Direct medical costs were measured as the sum of payments made by insurer and patients, OTC drugs and other costs, such as medical equipment. In the case of direct non-medical costs, transportation and caregiver costs were estimated. Productivity loss was calculated for patients up to 65 years of age. Intangible costs were excluded in this study due to their subjectivity [18].

This study limited musculoskeletal disease to "M00-M99" as defined by the International Classification of Diseases (ICD) 10 [21]. Therefore, injury due to trauma was excluded. Musculoskeletal disease was further subdivided into 11 categories according to the Korean classification of 298 diseases [22]: inflammatory polyarthropathies (M05-M14), arthrosis (M15-M19), acquired deformities of the limb(s) (M20-M21), other arthropathies (M00-M03, M22-M25), systemic connective tissue disorder (M30-M36), cervical or other intervertebral disorder (M50-M51), other dorsopathies (M40-M49, M53-M54), soft tissue disorder (M60-M79), disorder of bone density and structure disorders (M80-M85), osteomyelitis (M86), and other disorders resulting from musculoskeletal disease (M87-M99).

Because Korea has a social health insurance system and the NHIC is the exclusive insurer, NHIC claims data are nationally representative of medical care costs covered by the Korean insurance program [23,24]. However, NHIC claims data do not include non-covered costs such as elective services, caregiver costs, and over the counter (OTC) drugs costs. In order to supply this missing information, data from the Korea Health Panel were used in this study.

The Korea Health Panel study was conducted in 2007 through collaboration between the Korea Institute for Health and Social Affairs and the NHIC to determine the health service use behaviour of patients and medical expenditures [25]. The survey sample was extracted using two-stage cluster stratified sampling from the 2005 Korean census. A pilot test was conducted in 2007, and the first study wave was conducted in 2008. The survey was composed of about 200 questionnaires and interviews conducted by trained interviewers. The survey was performed on household, individual, and case bases. The household study included questions about the general characteristics, living expenses, purchase of pharmaceutical products, and private health insurance and premiums of the household. The individual study considered demographic characteristics of the subjects. The case-based study included those with chronic diseases, inpatients, outpatients, and emergency service utilization. In the case of chronic disease, survey subjects were asked whether they had a chronic disease. If they responded positively, then they were asked whether they had been diagnosed by physician.

In our study, the data sources were utilized as follows. First, the first round of the Korea Health Panel data was used to determine the prevalence of musculoskeletal disease. Musculoskeletal disease patients were defined as those who responded that they had a chronic musculoskeletal disease that had been diagnosed by a doctor. In the Korea Health panel, diseases are coded according to the Korean classification of 298 diseases. Patients diagnosed with any of the 11 musculoskeletal diseases in this classification were defined as musculoskeletal patients (inflammatory polyarthropathies (M05-M14), arthrosis (M15-M19), acquired deformities of the limb(s) (M20-M21), other arthropathies (M00-M03, M22-M25), systemic connective tissue disorders (M30-M36), cervical or other intervertebral disorders (M50-M51), other dorsopathies (M40-M49, M53-M54), soft tissue disorders (M60-M79), disorders of bone density and structure disorders (M80-M85), osteomyelitis (M86), and other disorders associated with musculoskeletal disease (M87-M99)). Some subjects in the survey had multiple chronic diseases. Subjects were diagnosed as musculoskeletal patients if they had at least one musculoskeletal disease. The prevalence rate was measured as the proportion of musculoskeletal patients among the participants of the Korea Health Panel according to age group (0-19 years old, 20-64 years old and ≥65 years old). The population of Korea was multiplied by the prevalence rate to estimate the numbers of patients with musculoskeletal diseases.

Among the economic cost, medical care costs paid by insurer were estimated using NHIC statistics for the year 2008 [8]. Additionally, copayment costs and non-covered care costs were estimated using the first round of the 2008 Korea Health Panel data and were estimated as patient costs. OTC drugs costs and other costs such as medical equipment costs were also estimated using the first round of the 2008 Korea Health Panel data. In the breakdown of direct non-medical costs, transportation costs were estimated using the first round of the 2008 Korea Health Panel data, and caregiver costs were estimated from NHIC statistics. Indirect costs were composed of premature death costs and productivity losses due to hospital admission and outpatient visits. The total numbers of admission days were acquired from NHIC statistics, and the number of outpatient visits was estimated from the Korea Health Panel data. Cause of death data as reported by the National Statistical Office (NSO) in 2008 were used to estimate the cost of premature death. Likewise, the total population figure used in this study was the NSO's estimated population for 2008 [15]. All measured costs were calculated in US dollars (1$ = 1104.7 won) [26].

Inpatient, outpatient, and emergency service due to musculoskeletal disease according Korea Health Panel data were analysed to estimate the payment by patients. Some patients had multiple reasons for using these services, but if at least one of the patient diagnoses was musculoskeletal disease, these visits were considered in this study. The costs paid by patients were adjusted by the number of chronic diseases reported. However, the cost of each musculoskeletal disease was classified according to the major diagnosis. The study period for the first round of the Korea Health Panel was 192.1 days and was adjusted to annual cost. The total cost to the Korean population was estimated as the total population compared to the number of subjects surveyed in the Korea Health Panel. Also, the cost of OTC drugs and other costs were determined from the Korea Health Panel data. Other costs included the cost of medical equipment, herbal medicine expenditures, health supplement food, and sanitary aid associated with musculoskeletal disease. OTC drugs and other costs were surveyed by household in the Korea Health Panel. Musculoskeletal disease patients were identified, and household OTC costs and other costs were analysed from this data. Finally, costs were adjusted according to the number of persons in each household and the number of chronic diseases in the musculoskeletal patients. 

Cost paid by the patient = (inpatient costs + outpatient costs + emergency service costs)*(365/192.1)*(the population of Korea in 2008/the number of subjects surveyed).

OTC drugs costs = (OTC drugs costs for musculoskeletal disease) * (365/192.1) * (the population of Korea in 2008/the number of subjects surveyed).

Other costs = (medical equipment costs + herbal medicine expenditure in market or pharmacy cost + health supplement food costs + sanitary aid costs)*(365/192.1)*(the population of Korea in 2008/the number of subjects surveyed).

In the analysis of direct costs, transportation costs were estimated using data from the Korea Health Panel study. The frequencies of inpatient and outpatient service use due to musculoskeletal disease were determined, and the average cost of transportation for musculoskeletal disease patients was estimated. The average cost of using medical services was estimated at $0.58 per one way trip for outpatient service and $8.68 per one way trip for inpatient service.

Transportation Costs = inpatient transportation costs + outpatient transportation costs = (frequency of inpatients with musculoskeletal disease * average one-way trip cost of inpatients*2+ frequency of outpatients with musculoskeletal disease *average one-way trip cost of outpatients*2) *(365/192.1) *(the population of Korea in 2008/the number of subjects surveyed).

In the case of caregiver costs, the cost and percentage of patients who used a caregiver, including an informal caregiver such as a relative or friend, were estimated from data from the Korea Health Panel study, and the admission day was estimated using NHIC statistics. The average cost per day of paid caregivers for patients with musculoskeletal disease was $45, and the percentage of patients that used a caregiver was 74%.

Caregiver costs = (days of hospitalization*average caregiver cost per day in 2008($45))* (the percentage of caregiver utilization in musculoskeletal patients).

Indirect costs were divided into productivity loss costs and premature death costs. Costs due to loss of productivity were calculated from productivity loss due to outpatient and inpatient visits for patients between 20 and 64 years of age. Average wage per day according to age group was estimated from Ministry of Labor statistics [27]. For inpatients, the total duration of hospitalization was estimated from NHIC statistics, and for outpatients, the number of outpatient visits per age group was estimated using data from the Korea Health Panel study. We regarded the productivity loss due to outpatient visits as 1/3 that of the productivity loss for inpatients per day [19].

Productivity loss = productivity loss of inpatients and outpatients = days of hospitalization * average wage per day + frequency of outpatient visits * average wage per day*(1/3).

In order to estimate the cost of premature death, mortality due to musculoskeletal disease was determined for each age and sex group using cause of death reports generated by the National Statistical Office (NSO) [28]. Lost future wages of the deceased were acquired from Ministry of Labor statistics and discounted by five percent per year to obtain the present value of forgone income [20]. Future income loss was calculated through 65 years of age. Finally, mortality due to musculoskeletal disease was multiplied by the present value of future income.

Premature death costs = present value of future income according to age & sex * frequency of mortality according to age &sex group.

This study used public data from the NHIC, the Korea Health Panel and the NSO which do not have personal identifiers, and data publishing was approved by each organization's ethics committee. This survey conformed to the Declaration of Helsinki as well as to local legislation. The data were collected and analysed using Microsoft Excel and SAS 9.2 (SAS Institute, Inc., Cary, NC).

## Results

Table 1 shows the estimated prevalence of musculoskeletal disease determined using the Korea Health Panel. The prevalence of musculoskeletal disease in male children and adolescents under 20 years old was 334.1 per 100,000 persons, and the prevalence for females of the same age was 455.0 persons per 100,000 persons. Prevalence increased with age. The prevalence in females was 12,605.3 persons per 100,000, and 5,697.3 persons per 100,000 in males for those 20-64 years of age. In those 65 years or older, the prevalence of musculoskeletal disease was 25092.3 persons in males and 56,657.2 persons per 100,000 in females. The total number of patients with musculoskeletal disease was 5.4 million, and the prevalence of musculoskeletal disease was estimated to be 11,212.7 per 100,000 persons (Table 1).

**Table 1:** Prevalence per 100,000 persons and numbers of musculoskeletal diseases patients by age group in male and female in Korea, 2008

		Male			Female	
	
	Population^*^	**Prevalence**^**†**^	Patients^*^	Population^*^	**Prevalence**^**†**^	Patients^*^
						
0-19 years old	6167.7	334.1	20.6	5567.1	455.0	25.3
20-64 years old	16216.1	5697.3	923.9	15640.0	12605.3	19714.6
≥65 years old	2032.2	25092.3	509.9	2983.8	56657.2	16905.6
Total	24415.9	6412.8	1454.4	24190.9	15818.8	36873.5

*Unit: thousand persons

^†^Unit: per 100,000 persons.

The five most costly musculoskeletal diseases are noted in Table 2. The most costly musculoskeletal disease was other dorsopathies (M40-49& M53-M54), costing a total of 1.42 billion dollars (536.2 million dollars for males and 855.3 million dollars for females). The next most costly musculoskeletal diseases were disc disorders (1.32 billion dollars), arthrosis (1.24 billion dollars), soft tissue disorders (1.11 billion dollars) and inflammatory polyarthropathies (345 million dollars). In the case of arthrosis, female patients accounted for 75% of the total cost of this disorder (9.33 million dollars), which is a much higher proportion than that for other musculoskeletal diseases.

**Table 2:** Economic burden and prevalence of the five most costly musculoskeletal disease, 2008

	male		female		total	
	
	prevalence^*^	**cost**^**†**^	prevalence^*^	**cost**^**†**^	prevalence^*^	**cost**^**†**^
Other dorsopathies	1808.5	563.2	3367.6	855.3	2604.1	1418.5
Disc disorders	1443.5	535.8	2213.2	781.9	1836.3	1317.7
Arthrosis	1609.4	307.5	7754.2	932.9	4745.1	1240.4
Soft tissue disorders	1161.4	381.1	2404.3	731.8	1795.7	1112.9
Inflammatory polyarthropathy	613.9	113.8	883.7	231.0	751.6	344.8

*Unit: per 100,000 persons

^†^Unit: million dollars.

Direct medical care costs, direct non-medical care costs, and indirect costs are shown in Table 3. Direct medical care costs accounted for the highest proportion of costs. Among direct medical care costs, costs paid by insurer were the highest at 2.47 billion dollars, and costs paid by patients, comprised of co-payments and non-covered care costs, followed at 1.68 billion dollars. Other costs, including medical equipment and health supplement food, totalled 25 million dollars, and OTC drugs costs were 11 million dollars. The total cost of direct medical care was 4.18 billion dollars. Additionally, the costs associated with caregivers and transportation were 269 million dollars and 69 million dollars, respectively. In total, direct costs were estimated as 4.52 billion dollars.

**Table 3:** Direct and indirect costs of musculoskeletal disease in Korea, 2008

		Direct costs								Indirect costs		**Total costs**^**†**^
Age group	Population	Direct medical care costs					Direct non-medical care costs		Total direct costs			
	(thousand persons)	Paid by insurer	Paid by patient	OTC drugs costs	Other costs	Total direct medical care costs^*^	Transportation	Caregiver costs		Lost productivity	Cost of premature death	
Male												
0-19 years	6,168	17,954	39,549	47	97	57,647	506	2,901	61,054	0	640	61,694
20-64 years	16,216	443,446	361,346	2,092	4,760	811,644	11,144	66,877	889,666	753,139	31,248	1,674,052
≥65 years	2,032	368,355	169,309	2,099	1,879	541,643	7,518	43,610	592,771	0	0	592,771
Male Cost	24,416	829,756	570,204	4,239	6,736	1,410,934	19,169	113,388	1,543,491	753,139	31,888	2,328,517
Female												
0-19 years	5,567	35,514	22,019	43	83	57,658	474	3,992	62,125	0	2,505	64,630
20-64 years	15,640	877,142	625,749	4,761	11,667	1,519,320	22,484	92,053	1,633,856	1,530,437	45,028	3,209,321
≥65 years	2,984	728,612	459,014	2,377	6,494	1,196,497	26,857	60,027	1,283,382	0	0	1,283,382
Female Cost	24,191	1,641,268	1,106,783	7,181	18,244	2,773,475	49,815	156,073	2,979,363	1,530,437	47,533	4,557,333
Total Cost	48,607	2,471,024	1,676,987	11,419	24,980	4,184,409	68,984	269,461	4,522,854	2,283,575	79,421	6,885,850

OTC drugs: Over the counter drugs, Unit: thousand dollars

*Sum of costs paid by insurer + costs paid by patients + OTC drugs costs+ other costs

^†^Sum of direct costs + indirect costs.

Indirect costs were composed of productivity loss costs and costs due to premature death. The cost due to productivity loss was estimated to be 2.28 billion dollars. The total number of deaths due to musculoskeletal disease was 1,699 according to NSO statistics (male: 513, female: 1,156). The leading cause of death was disorders of bone density and structures including osteoporosis, which affected 676 patients. Mortality rates were higher in females 65 years and older (565 people). The cost due to premature death was 79 million dollars, and systemic connective tissue disorders accounted for the highest proportion of these costs (40 million dollars).

When costs were compared according to age group and sex, costs for female adults aged 20-64 were the highest (3.21 billion). However, when compared with the population, the burden of musculoskeletal disease was highest for aged females ($430 per person), followed by aged males ($291), female adults ($205), and male adults ($103). The proportions of costs for male and female patients were 33.8% and 66.2%, respectively, and those of direct medical care costs, direct non-medical care costs, and indirect costs were 60.8%, 4.9%, and 34.3%, respectively. Total costs, which included direct costs and indirect costs, were 6.89 billion dollars (Table 3).

## Discussion

In this study, the prevalence of musculoskeletal disease was estimated to be 11,212.7 persons per 100,000 and the three most costly musculoskeletal diseases were other dorsopathies, disc disorders, and arthrosis. Direct medical costs accounted for more than half of the total costs, and costs paid by insurer were 59% of the direct medical costs. The costs paid by female adults aged 20-64 were highest, but the economic burden per person was heaviest for aged females (65 years and older group). Males accounted for only one-third of the total costs.

The prevalence of musculoskeletal disease in this study is relatively low compared to those in previous studies in Korea [11,12]. One study in Korea estimated the population prevalence of musculoskeletal disease in adults to be 19,720 per 100,000 according to annual self-reported prevalence rate, and 16,340 per 100,000 according to annual physician diagnosed prevalence rates. Furthermore, the prevalence of musculoskeletal disease in females 65 years or older was estimated at 73,100 per 100,000 [11]. The differences in these results can be attributed to differences in the age groups studied. In this study, the prevalence rate was calculated out of the total population. The physician-diagnosed prevalence rate for adults would then be 14989.6 per 100,000, a result similar to that found in previous studies [11].

Differences in case definition and the methodology used to estimate prevalence could also result in differences in the estimation of disease prevalence. Hur et al. [11] defined musculoskeletal disease as being comprised of the main musculoskeletal diseases of osteoarthritis, rheumatoid arthritis, osteoporosis and herniated discs. Self-reported case defined by the patients of who have disease more than three months during last year or currently suffering. Patients who have self-reported musculoskeletal disease and diagnosed by physician were considered physician diagnosed patients. In this study, we considered all musculoskeletal diseases, and cases were defined as those patients with a musculoskeletal chronic disease which had been diagnosed by a physician. Additionally, the prevalence rate in this study was estimated from the proportion, while the previous study used weighting factors to estimate the prevalence [11].

Musculoskeletal disease prevalence varies in other studies. For example, one study in the United Kingdom reported that 14.5% of adults had symptoms of musculoskeletal disease in 2003 [29]. Another study estimated the prevalence of musculoskeletal disease to range from 6.6% to 20.7% [30]. Moreover, prevalence rates vary according to the type of musculoskeletal disease. For example, the global prevalence of arthritis has been reported to range from 0.5% to 36%. This large range results from differing case definitions and investigation methods, as well as differences in race and country [11].

Furthermore, variation in estimates of the economic burden of musculoskeletal disease is due to differences in the types of diseases included as well as the cost calculation methods used. For example, in Canada, direct and indirect costs associated with musculoskeletal disease were estimated to be 5.6 billion dollars and 13.5 billion dollars, respectively. Total economic costs in 1994 were estimated to be 19.2 billion, about 3.4% of the Canadian gross domestic product (GDP) [7]. In the Canadian study, musculoskeletal disorders included injuries, fractures, and neoplasms of musculoskeletal origin. Direct costs also included the cost of nursing home admissions. However, transportation costs were excluded. In addition, future income was discounted at 6% annually. In Sweden, the economic cost of musculoskeletal disease, including rheumatoid arthritis, osteoarthritis, back diagnoses and other musculoskeletal system diseases, was 52,762 million Swedish Kronor in 1994 [31]. This represented about 3.14% of the Swedish GDP in 1994 [32]. In the Swedish study, costs included inpatient care, outpatient care and loss of productivity. In the USA, the direct cost of musculoskeletal disease was estimated at $510 billion and the indirect cost was $339 billion which were 7.7% of the GDP [8]. In USA study, musculoskeletal injuries were included in the musculoskeletal disease entity, and the cost of musculoskeletal disease was measured by the individual patient. Total costs were estimated as the sum of individual persons' costs rather than the sum of cost components. Direct costs due to disease and indirect costs due to earning loss were measured, while costs due to premature mortality were not considered. The total medical expenditures of patients with musculoskeletal conditions were $5,824. Additionally, incremental expenditures of musculoskeletal patients were $1,789 compared to those subjects with similar characteristics but without a diagnosis of musculoskeletal disease. The average earning loss of musculoskeletal patients was $4,616 and the difference was $1,506 with those who did not have musculoskeletal diseases. Total direct and indirect costs were 4.6% and 3.1% of the GDP, respectively, and incremental direct and indirect costs were 1.4% and 1.0% [7]. Therefore, the estimated economic burden of musculoskeletal disease differs according to study design and methods.

In this study, direct costs were 65.7% of total costs and the total cost of musculoskeletal disease was about 0.7% of the Korean GDP [10]. These results vary from previous studies due to differences in study design and country of study. For instance, costs due to musculoskeletal injuries, neoplasms of musculoskeletal origin and congenital malformations of the musculoskeletal system such as spina bifida were not measured in this study. Additionally, this relatively low proportion may be related to the low levels of health care expenditures in Korea [33].

When compared with other Korean studies using a similar study design, the total cost of musculoskeletal disease in 2002 ($6.89 billion) was lower than that of cancer ($9.4 billion) [20]. The costs of disc disorders and arthrosis were 4.83 times and 4.54 times higher than that of allergic rhinitis, respectively [19]. In comparison, the economic burden of cancer in Korea was 1.72% of the GDP in 2002.

Though the prevalence and economic burden of musculoskeletal disease vary, the fact that the prevalence of musculoskeletal disease was found to be higher in the aged and in females in our study sample is consistent with the results of most other studies [11,12,29,34]. Korea has a rapidly aging population due to a decrease in birth rate and an increase in life expectancy. The percentage of the population 65 years or older will increase from 10.3% in 2008 to 15.6% in 2020 and to 38.2% in 2050 [10]. The prevalence and economic burden of musculoskeletal disease will increase in the coming years. The cost of musculoskeletal disease is predicted to increase by 8 billion dollars in 2020 and by 9.2 billion dollars in 2050 [10], without taking into account additional cost increases resulting from income growth or technological advances. Thus, musculoskeletal disease will impose a heavier economic burden on Korea in the near future.

In this study, other dorsopathies (M40-M49, M53-M54) is the most costly disease entity. This includes the low back pain category (M45-M48, M54 except M54.2) of the Global Burden of Disease Study and also includes deforming dorsopathies and dorsalgia [3]. Among dorsopathies, only disc disorders (M50-M51) are excluded from the category of other dorsopathies. The fact that this is a broad disease category could be behind these high costs.

In this study, the classification of disease was based on Korean standards. For example, though the definition of arthrosis was consistent with that used in the Global Burden of Disease Study [3], apart from other dorsopathies, inflammatory polyarthropathies include various disease categories such as rheumatoid arthritis, gout, and others. This inconsistency could be a limitation for an international comparison of individual diseases.

Several additional limitations should be noted in this study. First, the Korea Health Panel which was used to estimate components of cost and prevalence does not include weighting factors to estimate national statistics, therefore the accuracy of the cost components using Korean Health Panel could be limited. Second, this study did not consider the cost of disability due to disease or other intangible costs. Some musculoskeletal diseases such as osteoarthritis could result in disability or sequelae, so could result in reduced quality of life. Hence, if these costs were considered, the economic burden of musculoskeletal disease would be greater [35]. Additionally, we presumed that costs due to premature death and productivity loss were not incurred by the aged (those 65 years or older). However, these results may have varied with a different assumption [20]. Moreover, when using the NHIC statistics to estimate costs according to sex, the proportion of costs according to the Korea Health Panel study was used. Another limitation is that the costs paid by patients did not distinguish between copayment costs and non-covered care costs. Therefore, it is difficult to determine actual non-covered care costs of musculoskeletal disease, which are a public concern in Korea.

## Conclusions

In conclusion, this study estimated the economic burden of musculoskeletal disease including non-covered care costs, OTC drugs costs, and other costs such as medical equipment expenditures, which were not sufficiently considered in previous studies. Furthermore, this study is the first to estimate the economic burden of all musculoskeletal diseases in Korea. In this study, the estimated population prevalence of musculoskeletal disease was 11212.7 persons per 100,000, and the economic burden was 2.33 billion in males and 4.56 billion in females, totalling 6.89 billion dollars and 0.7% of the Korean GDP. The average economic burden of musculoskeletal disease per person was highest in aged females. As the Korean population ages, the economic burden of musculoskeletal disease will continue to increase. Therefore, adequate policy measures that minimize the cost of musculoskeletal disease will be required in the near future.

## Competing interests

The authors declare that they have no competing interests.

## Authors' contributions

SJY planned and supervised all aspects of this study. IHO interpreted the data and prepared a draft manuscript. EJK participated in the statistical analysis, and YAK and SHK contributed to the study and manuscript revision. All authors read and approved the final manuscript.

## Pre-publication history

The pre-publication history for this paper can be accessed here:

http://www.biomedcentral.com/1471-2474/12/157/prepub

## References

[B1] Woolf AD Pfleger B Burden of major musculoskeletal conditions Bull World Health Organ 2003 81 646 656 14710506 PMC2572542

[B2] Lopez AD Mathers CD Ezzati M Jamison DT Murray CJL Global and regional burden of disease and risk factors, 2001: systematic analysis of population health data The Lancet 2006 367 1747 1757 10.1016/S0140-6736(06)68770-9 16731270

[B3] World Health Organization The global burden of disease: 2004 update 2008 Geneva

[B4] Mili F Helmick CG Zack MM Prevalence of arthritis: analysis of data from the US behavioral risk factor surveillance system, 1996-99 J Rheumatol 2002 29 1981 1988 12233896

[B5] Phua H Chua A Ma S Heng D Chew S Singapore's burden of disease and injury 2004 Singapore Med J 2009 50 468 478 19495514

[B6] Yoon SJ Bae SC Lee SI Chang H Jo HS Sung JH Park JH Lee JY Shin Y Measuring the burden of disease in Korea J Korean Med Sci 2007 22 518 523 10.3346/jkms.2007.22.3.518 17596664 PMC2693648

[B7] Coyte PC Asche CV Croxford R Chan B The economic cost of musculoskeletal disorders in Canada Arthritis Rheum 1998 11 315 325 10.1002/art.1790110503 9830876

[B8] American academy of Orthopedic Surgeons The burden of musculoskeletal disease in the United States 2008 Rosmond

[B9] National Health Insurance Corporation, Health Insurance Review & Assessment Service National health insurance statistical yearbook 2009 Seoul in Korean

[B10] The Bank of Korea Quarterly National Accounts 2009 Seoul In Korean

[B11] Hur NW Choi CB Uhm WS Bae SC The prevalence and trend of arthritis in Korea: results from Korea National Health and Nutrition Examination Surveys J Korean Rheum Assoc 2008 15 11 26 in Korean 10.4078/jkra.2008.15.1.11

[B12] Choi HJ Han WJ Im JS Baek HJ The prevalence and clinical features of musculoskeletal diseases in Incheon: results from chronic disease management surveys J Korean Rheum Assoc 2009 16 281 290 in Korean 10.4078/jkra.2009.16.4.281

[B13] Bae SC The current status of surveys on prevalence of rheumatic diseases in Korea J Korean Rheum Assoc 2010 17 1 3 in Korean 10.4078/jkra.2010.17.1.1

[B14] Kim DW Ahn CW Nam SY Prevalence of obesity in Korea Obes Rev 2005 6 117 121 10.1111/j.1467-789X.2005.00173.x 15836462

[B15] Population projections for Korea http://kosis.kr/gen_etl/start.jsp?orgId=101&tblId=DT_1B01001&conn_path=K1 in Korean

[B16] Cooper NJ Economic burden of rheumatoid arthritis: a systematic review Rheumatology (Oxford) 2000 39 28 33 10.1093/rheumatology/39.1.28 10662870

[B17] McIntosh E The cost of rheumatoid arthritis Rheumatology 1996 35 781 790 10.1093/rheumatology/35.8.781 8761194

[B18] Tarricone R Cost-of-illness analysis. What room in health economics? Health Policy 2006 77 51 63 10.1016/j.healthpol.2005.07.016 16139925

[B19] Kim SY Yoon SJ Jo MW Kim EJ Kim HJ Oh Ih Economic burden of allergic rhinitis in Korea Am J Rhinol Allergy 2010 24 e110 e113 21244726 10.2500/ajra.2010.24.3513

[B20] Kim SG Hahm MI Choi KS Seung NY Shin HR Park EC The economic burden of cancer in Korea in 2002 Eur J Cancer Care (Engl) 2008 17 136 144 10.1111/j.1365-2354.2007.00818.x 18302650

[B21] International Statistical Classification of Diseases and Related Health Problems 10^th ^Revision Version for 2007 http://apps.who.int/classifications/apps/icd/icd10online/

[B22] Classification of 298 diseases http://www.nhic.or.kr/infopub/dc/down/symtom.hwp in Korean

[B23] Lee SY Chun CB Lee YG Seo NK The National Health Insurance system as one type of new typology: The case of South Korea and Taiwan Health Policy 2008 85 105 113 10.1016/j.healthpol.2007.07.006 17709152

[B24] Kang HY Yang KH Kim YN Moon SH Choi WJ Kang DR Park SE Incidence and mortality of hip fracture among the elderly population in South Korea: a population-based study using the national health insurance claims data BMC Public Health 2010 10 230 10.1186/1471-2458-10-230 20438644 PMC2874780

[B25] Korea Health Panel http://www.khp.re.kr/english/about_01.jsp

[B26] Average exchange rate of KEB bank http://fx.keb.co.kr/FER1201C.web in Korean

[B27] Ministry of Labor 2008 the survey report on labor conditions by employment type 2009 Seoul in Korean

[B28] National Statistical Office The cause of death statistics of 2008(raw data) 2009 Seoul In Korean

[B29] Clarke AM Symmons DPM The burden of rheumatic disease Medicine 2006 34 333 335 10.1053/j.mpmed.2006.06.007

[B30] Jordan K Clarke AM Symmons DPM Fleming D Porcheret M Kadam UT Croft P Measuring disease prevalence: a comparison of musculoskeletal disease using four general practice consultation databases Br J Gen Pract 2007 57 7 17244418 PMC2032694

[B31] Jonsson D Husberg M Socioeconomic costs of rheumatic diseases. Implications for technology assessment Int J Technol Assess Health Care 2000 16 1193 1200 10.1017/S0266462300103228 11155838

[B32] Statistics Sweden http://www.scb.se/Pages/ProductTables____22932.aspx

[B33] Organisation for economic co-operation and development OECD Health at a Glance 2009: OECD indicators 2009 Paris

[B34] Rat AC Boissier MC Rheumatoid arthritis: direct and indirect costs Joint Bone Spine 2004 71 518 524 10.1016/j.jbspin.2004.01.003 15589432

[B35] Lubeck DP The costs of musculoskeletal disease: health needs assessment and health economics Best Pract Res Clin Rheumatol 2003 17 529 539 10.1016/S1521-6942(03)00023-8 12787516

